# Mechanism(s) of action underlying the gastroprotective effect of ethyl acetate fraction obtained from the crude methanolic leaves extract of *Muntingia calabura*

**DOI:** 10.1186/s12906-016-1041-0

**Published:** 2016-02-24

**Authors:** Zainul Amiruddin Zakaria, Tavamani Balan, Ahmad Khusairi Azemi, Maizatul Hasyima Omar, Norhafizah Mohtarrudin, Zuraini Ahmad, Muhammad Nazrul Hakim Abdullah, Mohd. Nasir Mohd. Desa, Lay Kek Teh, Mohd. Zaki Salleh

**Affiliations:** Department of Biomedical Sciences, Faculty of Medicine and Health Sciences, Universiti Putra Malaysia, Serdang, Selangor 43400 Malaysia; Integrative Pharmacogenomics Institute (iPROMISE), Level 7, FF3 Building, Universiti Teknologi MARA, 42300 Puncak Alam, Selangor Malaysia; Halal Product Research Institute, Universiti Putra Malaysia, Serdang, 43400 Selangor Malaysia; Phytochemistry Unit, Herbal Medicine Research Centre, Institute for Medical Research, Jalan Pahang, 50588 Kuala Lumpur, Malaysia; Department of Pathology, Faculty of Medicine and Health Sciences, Universiti Putra Malaysia, Serdang, Selangor 43400 Malaysia

**Keywords:** *Muntingia calabura*, Fraction, Gastric ulcer, Antisecretory, Antioxidant, Nitric oxide, Sulfhydryl compound, Prostaglandin, Quercetin, Gallic acid

## Abstract

**Background:**

*Muntingia calabura* L. (family Muntingiaceae), commonly known as Jamaican cherry or *kerukup siam* in Malaysia, is used traditionally to treat various ailments. The aim of this study is to elucidate the possible underlying gastroprotective mechanisms of ethyl acetate fraction (EAF) of *Muntingia calabura* methanolic leaves extract (MEMC).

**Methods:**

MEMC and its fractions were subjected to HPLC analysis to identify and quantify the presence of its phyto-constituents. The mechanism of gastroptotection of EAF was further investigated using pylorus ligation-induced gastric lesion rat model (100, 250, and 500 mg/kg). Macroscopic analysis of the stomach, evaluation of gastric content parameters such as volume, pH, free and total acidity, protein estimation, and quantification of mucus were carried out. The participation of nitric oxide (NO) and sulfhydryl (SH) compounds was evaluated and the superoxide dismutase (SOD), gluthathione (GSH), catalase (CAT), malondialdehyde (MDA), prostaglandin E_2_ (PGE_2_) and NO level in the ethanol induced stomach tissue homogenate was determined.

**Results:**

HPLC analysis confirmed the presence of quercetin and gallic acid in EAF. In pylorus-ligation model, EAF significantly (*p* <0.001) prevent gastric lesion formation. Volume of gastric content and total protein content reduced significantly (*p* < 0.01 and *p* < 0.05, respectively), while free and total acidity reduced in the doses of 250 and 500 mg/kg (*p* <0.001 and *p* <0.05, respectively). EAF also augmented the mucus content significantly (*p* < 0.001). Pre-treatment with N-nitro-L-arginine methyl ester (L-NAME) or N-ethylmaleimide (NEM) reversed the gastroprotective activity of EAF. EAF treatment markedly ameliorated the SOD, GSH and CAT activity and PGE_2_ and NO level while attenuating MDA level, relative to the vehicle group.

**Conclusions:**

In conclusion, the underlying gastroprotective mechanisms of EAF could be associated with the antisecretory, participation of mucus, antiperoxidative, improvement of antioxidant status, modulation of NO and SH compounds, stimulation of PGE_2_ as well as presence of quercetin and gallic acid.

## Background

Gastric ulcer is one of the major gastrointestinal disorders that affect considerable number of people around the world, while growing in occurrence and prevalence globally [1]. Some authors refer to gastric ulcers as the new “plague” of the 21st century [2]. It has been projected that 14.5 million of the worldwide population are affected by gastric ulcers with a mortality rate of 4.08 million [3]. The pathophysiology of gastric ulcer is associated with the imbalance between aggressive and protective factors in the stomach. Gastric mucosal damage occurs when noxious factors “overwhelm” an intact mucosal defense, or weakening of the mucosal defensive mechanisms [4]. The noxious factors in this context include alcohol ingestion, acid and pepsin secretion, poor diet, stress, reactive oxygen species (ROS), the use of non-steroidal anti-inflammatory drugs (NSAIDs) and *Helicobacter pylori* infection [5, 6]. On the other hand, the key defense factors and mechanisms that afford mucosal defense include sufficient mucus secretion and mucosal blood flow, bicarbonate secretion, intact mucus barrier, prostaglandins, surface active phospholipids, increased levels of antioxidants, activity of anti-inflammatory compounds and adequate levels of nitric oxide (NO) [6–8].

Currently, the prevention and treatment of gastric ulcers has gained lots of interest and became and important challenge confronting medicine nowadays. To date, there are a few approaches used to prevent gastric ulceration, which include potentiation of the mucosal defense together with reduction of acid secretion and its neutralization, stimulation of gastric mucin synthesis, enhancement of antioxidant levels in the stomach, and inhibition of *Helicobacter pylori* growth [9]. Secretion of gastric acid is believed to be the central component of gastric ulcers despite the presence of many causative factors [7] and therefore, inhibition of gastric acid secretion tend to be the key therapeutic target for ulcer diseases [10]. On the other hand, another key factor in the pathogenesis of gastric ulcers is the production of reactive oxygen species (ROS). The production of ROS and a concomitant reduction of antioxidant capacity causes damage to the essential cell constituents, which are proteins, lipids and nucleic acids, resulting in the formation of toxic compounds and causes cell death due to their extreme reactivity [8, 11]. Therefore, controlling the ROS formation and gastric acid secretion are essential for the treatment of these pathologies [12].

The current medicinal treatment of gastric ulcers include acid blockers that reduce acid secretion, proton pump inhibitors, antibiotics to eradicate *Helicobacter pylori* and tissue lining protecting agents such as sucralfate and bismuth cholinergics [13, 14]. Eventhough these drugs have decreased the morbidity rates, but they are often associated with undesirable adverse effects such as hypersensitivity, impotence, arrhythmia, hematopoietic disorders, gynecomastia and antibiotic resistance in the long run [15, 16]. Furthermore, these available drugs also have high relapse rate, low efficacy in gastric ulcers treatment and are often costly [5, 9, 10]. Hence, there is a pressing need to discover a more effective and safe alternative therapies to treat gastric ulcers. In this context, the use of medicinal plants has gained interest and captured the attention of many researchers. Plant extracts can be valuable and serve as a new source of therapeutics in the treatment of gastric ulcers whereby antisecretory, cytoprotective and antioxidant activities, isolated or in combination, are the three main functions of a gastroprotective agent, which play the key role in gastric mucosal protection [17].

The plant *Muntingia calabura* L. (family Muntingiaceae), commonly known as Jamaican cherry or *kerukup siam* in Malaysia, is widely distributed throughout the warm areas of Asian region [18]. Several medicinal uses have been documented on various parts of this tree in East and Southeast Asia as well as tropical America. *Muntingia calabura's* leaves, flowers, barks and roots have been used as a folk remedy to treat headaches, fever and incipient cold. According to Peruvian folklore, the leaves are used to provide relief from gastric ulcers and to reduce swelling of the prostate gland [19]. Besides, they are also employed as antiseptic, antispasmodic, and antidyspeptic agent [20, 21].

On the other hand, *Muntingia calabura* is reported to possess a broad range of pharmacological activities, which have been proven scientifically. This include antitumor [20, 22], antibacterial [23], antinociception [19, 24, 25], anti-inflammatory, antipyretic [25], antioxidant and antiproliferative [26] activities exhibited by the leaves of *Muntingia calabura*, while several types of compounds have been isolated and identified from the leaves, roots and stem barks of *Muntingia calabura* [20–22, 27, 28]. Various phytochemicals have been detected in the leaves of *Muntingia calabura* such as flavonoids, saponins, tannins and triterpenes [29].

In our previous study, we have reported the gastroprotective activity of several fractions obtained from crude methanol extract of *Muntingia calabura* (MEMC) leaves against ethanol-induced gastric lesion in rats [30]. From our study, we have found that ethyl acetate fraction markedly ameliorate gastric ulceration and exerted the most effective protection as compared to the other fractions. Therefore, the present study was aimed to determine the mechanism of action underlying the prophylactic effect of the ethyl acetate fraction derived from MEMC against gastric lesions in rats.

Pylorus-ligation model used in this study is one of the most widely used models to study the effect of drugs on gastric acid and mucus secretion. Ulcers developed by ligating the pyloric end of the stomach are caused by increase in gastric hydrochloric acid (HCl) secretion and/or stasis of acid, leading to auto digestion of the gastric mucosa and breakdown of the gastric mucosal barrier [31]. Therefore, agents that are able to increase mucus secretion (cytoprotective) and/or reduce secretion of gastric aggressive factors such as pepsin and acid are effective gastroprotective agents [32]. On the other hand, the ethanol-induced ulcer model is useful for studying the efficacy of potential drugs or testing agents that have cytoprotective and/or antioxidant activities [33].

## Methods

### Chemicals

The chemicals used in this study are of analytical grades and had been prepared immediately before use. The following drugs were used: ranitidine (Sigma Aldrich, USA), absolute ethanol (Fischer Scientific, USA), N-ethylmaleimide (NEM) (Sigma-Aldrich, USA), N^G^-nitro-l-arginine methyl esters (L-NAME) (Sigma-Aldrich, USA), carbenoxolone (CBX) (Sigma-Aldrich, USA) and diethyl ether (Fischer Scientific, USA).

### Plant material

*Muntingia calabura* leaves were collected from their natural habitat in Shah Alam, Selangor, Malaysia, between May and August 2010. The plant was identified by a botanist from the Institute of Bioscience (IBS), Universiti Putra Malaysia (UPM), Serdang, Selangor. A voucher specimen, SK 2466/14, has been deposited at the UPM IBS Laboratory of Natural Products Herbarium.

### Extraction and fractionation of *Muntingia calabura* leaves

Method described by Zakaria et al. [26] and Sufian et al. [28] was employed to prepare the crude extract of *Muntingia calabura* leaves and its fractions, respectively. Five hundred grams of matured leaves were air-dried at room temperature (27 *±* 2 °C) for 1–2 weeks and ground into fine powder. Methanol (MeOH) was used as the solvent for extraction. The powder was soaked in MeOH at a ratio of 1:20 (w/v) for 72 h. The mixture was filtered using filter funnel, cotton and Whatman No. 1 filter paper. The soaking and filtration were repeated on the residue for twice. The filtrate collected from each extraction was pooled and evaporated in a rotary evaporator at 40 °C under reduced pressure to obtain methanol extract of *Muntingia calabura* (MEMC). The dried crude extract was suspended in MeOH and distilled water (dH_2_O) water in the ratio of 1:2 to afford an aqueous MeOH solution. The mixture was sequentially partitioned with different solvents, which were petroleum ether and ethyl acetate, yielding petroleum ether fraction (PEF), ethyl acetate fraction (EAF). The fractions were filtered and evaporated to dryness under vacuum using rotary evaporator. MEMC, PEF and EAF were subjected to HPLC to quantify the compound of interest, which could be associated with the extract’s gastroprotective effect.

### Identification and quantification of phytoconstituents present in EAF by HPLC

Method described by Zakaria et al. [34] with slight modifications was adapted to carry out the HPLC analysis of EAF. Briefly, 10 mg of sample was suspended in 1 ml methanol. The solutions were filtered through a filter cartridge (pore size of 0.45 μm) prior to use. The sample was analysed using a HPLC system (Waters Delta 600 with 600 Controller) with photodiode array detector (PDA) (Waters 996). A C_18_ column (4.6 mm i.d. × 250 mm) packed with 5 μm diameter particles was used. The mobile phase was water containing 0.1 % formic acid (A) and acetonitrile (B). Initial conditions were 85 % A and 15 % B with a linear gradient reaching 25 % B at *t* = 12 min. This condition was maintained for 10 min. B was reduced back to 15 %, the initial condition, and was maintained until *t* = 35 min. At *t* =25 min, the programme returned to the initial solvent composition. The flow rate was 1.0 ml/min, injection volume was 10 μl and the wavelength were 280 nm for gallic acid and 330 nm for quercetin. The column oven was set at 27 °C. Stock solutions of standards references were prepared in methanol at concentration 1 mg/ml. The chromatography peaks were confirmed by comparing its retention time with those of reference standards and by the respective UV spectra. Calibration curve for gallic acid was Y = 29562x + 102777 (R^2^ = 0.9969) and quercetin was Y = 43236x – 81458 (R^2^ = 0.999). All chromatography operations were carried out at ambient temperature and in triplicate. The HPLC analysis was carried out in the Laboratory of Phytomedicine, Medicinal Plants Division, Forest Research Institute of Malaysia (FRIM), Kepong, Malaysia.

### UHPLC–ESI analysis

The UHPLC system was performed on a Dionex 3000 UHPLC system acquired from Thermo Fisher Scientific (USA) that consisted of an autosampler equipped with a column oven, a tray compartment cooler, and a binary pump with built in solvent degasser. The chromatographic separation was performed on a BEH C18 UHPLC column, 100 mm x 2.5 μm, 1.7 μm (WATERS) at a flow rate of 0.3 mL/min. The mobile phases used were (A) 0.1 % formic acid in water and (B) 0.1 % formic acid in acetonitrile. The gradient started with 10 % mobile phase B, reaching 20 % mobile phase B at 5 min, 60 % mobile phase B at 17.0 min, at isocratic elution of 90 % B for 3 min. The gradient reached the initial conditions were held for 2 min as a re-equilibration step. The injection volume was 10 μL and the column temperature was maintained at 40 °C. The UHPLC system was coupled to a linear ion-trap-Orbitrap mass spectrometer Q Exactive from Thermo Fisher Scientific (U.S.A) equipped with an electrospray ionization (ESI) source. The mass detection was performed in a range of 150-1500 m/z. The ESI source was operated in negative ion mode under the following specific conditions: source voltage, 3.2 kV; sheath gas, 35 arbitrary units; auxiliary gas, 15 arbitrary unit; sweep gas, 10 arbitrary unit; and capillary temperature, 320 °C. Nitrogen (>99.98 %) was employed as sheath gas, auxiliary and sweep gas. Instrument control and data acquisition were performed with Chameleon 6.8 software and Xcalibur 2.2 software (Thermo Fisher Scientific).

### Animals

The experiments were performed on male Sprague Dawley rats (180–200 g; 8–10 weeks old). They were obtained from the Animal Unit, Faculty of Medicine and Health Sciences, UPM, Malaysia. The animals were kept in polypropylene cages with wood shaving, fed with standard pellet and allowed free access to water. They were kept in room temperature (27 ± 2 ^0^C; 70–80 % humidity; 12 h light/darkness cycle) in the Animal Holding Unit (UPM). Prior to all assays, the rats were fasted. Standard drugs and MEMC were administered orally (p.o.) by gavage with 8 % Tween 80 (10 ml/kg) as the vehicle. The use of animals in this study was approved by the Animal Care and Used Committee (ACUC) of UPM (Approval No: UPM/FPSK/PADS/BR-UUH/00474).

### Determination of the mechanism underlying the gastroprotective activity of EAF

#### Pyloric ligation

Method by Shay et al. [35] with slight modifications was employed to perform pyloric ligation. Rats were randomly divided into 5 groups, with six rats in each group. Group-I was the control group administered with 8 % Tween 80 (vehicle) orally (p.o), Group-II was the positive control administered with ranitidine at 100 mg/kg (p.o), while for Group-III,-IV and-V, rats were administered with EAF (100, 250 and 500 mg/kg, respectively). Pylorus ligation was performed on 48 h fasted rats 1 h after the administration of the test compounds. Under light anesthesia induced using ketamine HCl (100 mg/kg, intramuscular) and xylazine HCl (16 mg/kg, intramuscular), a 2 cm long mid-line abdominal incision was performed, just below the sternum. The pyloric portion of the stomach was gently mobilized and carefully ligated with a silk ligature around the pyloric sphincter in a tight knot. Care was taken while tying the knot to avoid interference with gastric blood supply. The abdominal incision was sutured, the skin was cleaned of any blood spots or bleeding and the animals were allowed to recover from anesthesia.

#### Assessment of gastric mucosal lesion

The animals were sacrificed 6 h after ligation by exposure to diethyl ether and cervical dislocation. The stomachs were removed, and the contents were drained out, collected, and centrifuged. The stomach was opened along the greater curvature to determine the lesion damage as described by Balan et al. [36]. The percentage protection was calculated using the following formula:

$$ \mathrm{Protection}\ \left(\%\right) = \frac{\left(\mathrm{U}\mathrm{A}\ \mathrm{control}\ \hbox{--}\ \mathrm{U}\mathrm{A}\ \mathrm{p}\mathrm{r}\mathrm{e}\hbox{-} \mathrm{treated}\ \mathrm{group}\right)}{\left(\mathrm{U}\mathrm{A}\ \mathrm{control}\right)} \times 100\% $$

#### Determination of volume, pH, free and total acidity of gastric content

The drained gastric content was centrifuged for 10 min at 2500 rpm to remove any solid debris. The volume and pH of the gastric juice was measured. The gastric juice was also subjected to free and total acidity estimation according to method described by Srivastava et al. [37]. Titration with 0.01 N NaOH with methyl orange reagent was carried out until the color of the solution became yellowish in order to determine the free acidity. The volume of alkali added was noted. Then, two to three drops of phenolphthalein was added to the solution. The solution was titrated until definite red tinges appear. The total volume of NaOH added was noted. This volume corresponds to total acidity. Acidity was calculated using the following formula:

$$ \mathrm{Acidity}=\frac{\mathrm{Volume}\ \mathrm{of}\ \mathrm{NaOH} \times \mathrm{normality}\ \mathrm{of}\ \mathrm{NaOH} \times 100}{0.1}\mathrm{m}\mathrm{e}\mathrm{q}/\mathrm{l} $$

#### Estimation of protein

The total protein content in gastric juice was estimated by Lowry’s method, adapted from Lowry et al. [38] using alkaline copper reagent and Folin’s reagent. The color developed was read at 660 nm. The protein content was calculated from the standard curve prepared with bovine serum albumin and protein concentration was expressed as mg/ml of gastric juice.

#### Estimation of gastric wall mucus content

Method described by Corne et al. [39] with slight modifications was employed to determine the gastric wall mucus content. The stomach was opened along the greater curvature, weighed, and immersed in 10 ml of 0.1 % Alcian blue (0.16 M sucrose in 0.05 M sodium acetate, pH 5.8) for 2 h. The stomach was then rinsed twice in 0.25 M sucrose solution (15 min each) to remove the excessive dye. The remaining dye that complexed with the gastric mucus was extracted with 0.5 M MgCl_2_. The glandular segment remained in this solution for 2 h with intermittent agitation for 1 min in every 30 min interval. The resultant blue extract was then shaken vigorously with an equal volume of diethyl ether until the formation of an emulsion. The resulting emulsion was centrifuged for 10 min at 3600 rpm. The absorbance of the aqueous layer was read at 580 nm using a spectrophotometer. The concentration of Alcian blue was calculated through a standard curve and the results were expressed in mg of Alcian blue/g of wet tissue.

#### Ethanol-induced gastric mucosal lesion in L-NAME or NEM pre-treated rats

The role of endogenous NO and the involvement of sulfhydryl (SH) compounds in the gastroprotective effect of EAF were evaluated using the method described by Takayama et al. [40]. Male rats were divided into 9 groups and pretreated (i.p.) with saline, L-NAME (N-nitro-L-arginine methyl ester, 70 mg/kg) an inhibitor of the NO synthesis or NEM (N-ethylmaleimide, 10 mg/kg) a SH compound blocker. Thirty minutes after the pre-treatment, the animals were administered (p.o.) vehicle (8 % Tween 80), carbenoxolone (100 mg/kg) or EAF (500 mg/Kg). Sixty minutes later, all groups received absolute ethanol (5 ml/kg, p.o) to induce gastric ulcers. All the rats were sacrificed 1 h after the administration of ethanol by exposure to diethyl ether and cervical dislocation. The stomach was removed and gastric damage was determined as described above. Since EAF exhibited a dose-dependent effect and exerted substantial protective action against gastric mucosa in the ethanol-induced gastric ulcer model, the highest effective dose (500 mg/kg) was used for this study.

### Biochemical analysis

#### Measurement of superoxide dismutase (SOD), glutathione (GSH) level and catalase (CAT) activity

Stomach tissues of the rats pre-treated with vehicle (8 % Tween 80), ranitidine (100 mg/kg) or EAF (100, 250 and 500 mg/kg) followed by ulcer induction using absolute ethanol for 1 h were used for the determination of SOD, GSH level and CAT activity. Gastric tissue was cut into pieces and the exact weight was recorded. The tissues were homogenized with a homogenizer using appropriate cold buffer and then were centrifuged at 10000 g for 15 min at 4 °C. The supernatants were used to determine the activities of CAT and levels of SOD, and GSH. The concentration of protein in the supernatants was measured by the Bradford method [41] using bovine serum albumin (BSA) as a standard. Levels of SOD, GSH and CAT were determined using the commercial assay kits according to the manufacturer’s instructions, respectively (Superoxide Dismutase Assay Kit, Glutathione Assay Kit and Catalase Assay Kit, Cayman Chemical Company, Ann Arbor, MI, US).

#### Measurement of malondialdehyde (MDA) level

MDA levels were measured in stomach tissues obtained from the ethanol-induced gastric ulcer. The stomach tissue was homogenized and centrifuged as described before and the supernatant was used for determination of MDA by using an enzyme-linked immunosorbent assay kit (USCN Life Science Inc., Atlanta, GA, USA). The optical densities were measured at 450 nm and the results were expressed as ng/mg protein.

#### Determination of prostaglandin E_2_ (PGE_2_)

PGE_2_ levels were determined in stomach tissues obtained from the ethanol-induced gastric ulcer. The supernatant of homogenized and centrifuged stomach tissue was used for determination of PGE_2_ by using Prostaglandin E_2_ Express EIA Kit (Cayman Chemical Company, Ann Arbor, MI, US). The optical densities were measured at 412 nm. The PGE_2_ concentrations were normalized by protein contents and the results were expressed as pg/mg protein.

#### Determination of NO level

 NO level in the gastric mucosa was evaluated as total nitrate/nitrite levels using Griess reagent [42]. In brief, the stomach homogenates were prepared in 50 mM potassium phosphate buffer (pH 7.8). The homogenates were centrifuged at 4000 rpm for 10 min at 4 °C. Fifty microliter of Griess reagent (0.1 % N-(1-naphthyl) ethylenediamide dihydrochloride, 1 % sulfanilamide in 5 % phosphoric acid) was added to 50 μL supernatant and the absorbance was measured at 540 nm after 10 min. Sodium nitrite was used as the standard in this assay to generate the standard curve.

### Statistical analysis

The results were expressed as mean ± standard error mean (SEM) and analyzed using One Way Analysis of Variance (ANOVA), followed by Dunnett’s *post hoc* test or the Newman-Keuls test. Results were considered significant when *p <* 0*.*05.

## Results

### Identification and quantification of gallic acid and quercetin

**Fig. 1:**
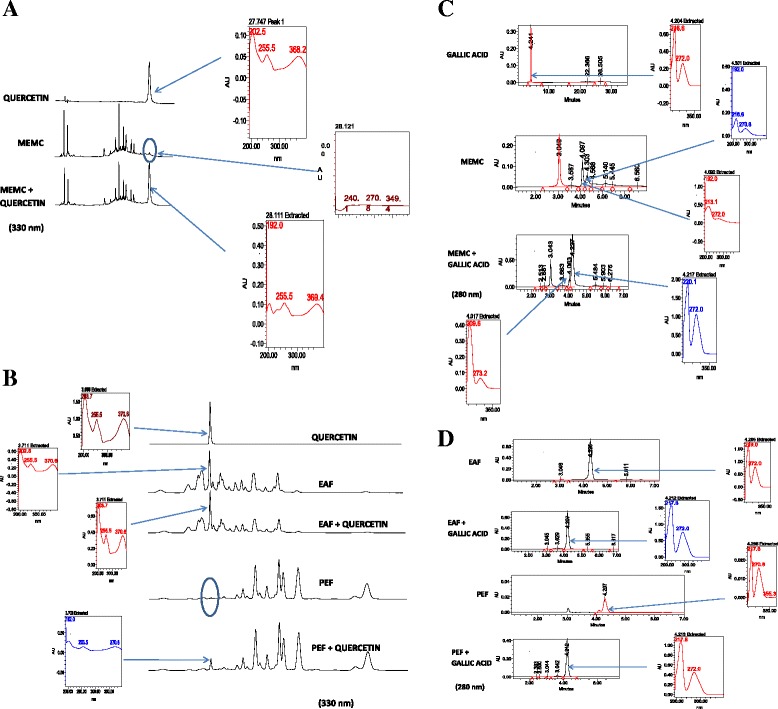
**a** and **b**: HPLC analysis of MEMC, PEF and EAF carried out at the wavelength 330 nm revealed the presence of the quercetin at λ_max_ 255.5-370.6 nm at RT 3.696 min. Spiking of quercetin with the extracts increased the peak area, corresponding to the same λ_max_ value of the compounds. **c** and **d**:HPLC fingerprinting of MEMC, PEF and EAF at the 280 nm wavelength revealed the presence of the gallicacid at λ_max_ 216.6-272.0 nm at RT 4.204 min. Spiking of gallic acid with the extracts increased the peak area, corresponding to the same λ_max_ value of the compounds

HPLC fingerprinting of MEMC, PEF and EAF revealed the presence of the gallic acid at λ_max_ 216.6–272.0 nm and quercetin at λ_max_ 255.5–370.6 nm (Fig. 1a and b). Spiking of these compounds in MEMC, PEF or EAF increased the peak area, corresponding to the same λ_max_ value of the compounds. The quantification result presented in Table 1 shows that EAF contains the highest amount of gallic acid (39.89 ± 0.96 mg/g extract) and quercetin (9.36 ± 0.29 mg/g extract) followed by MEMC and PEF.

**Table 1:** Gallic acid and quercetin’s composition in MEMC and its active fractions (PEF and EAF) at mg/1 g of extract. Results are expressed as mean ±  standard deviations (SDs) of three determinations

Compounds	MEMC	PEF	EAF
Gallic acid (mg/g)	11.97 ± 0.27	3.40 ± 0.01	39.89 ± 0.96
Quercetin (mg/g)	4.83 ± 0.16	8.81 ± 0.44	9.36 ± 0.29

### Identification of phenolic compounds in EAF

**Fig. 2:**
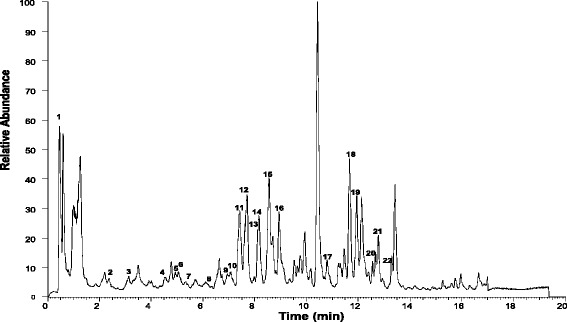
Total ion chromatography (TIC) of the indicatedEAF sample, obtained from the UHPLC instrument in negative ion mode

**Fig. 3:**
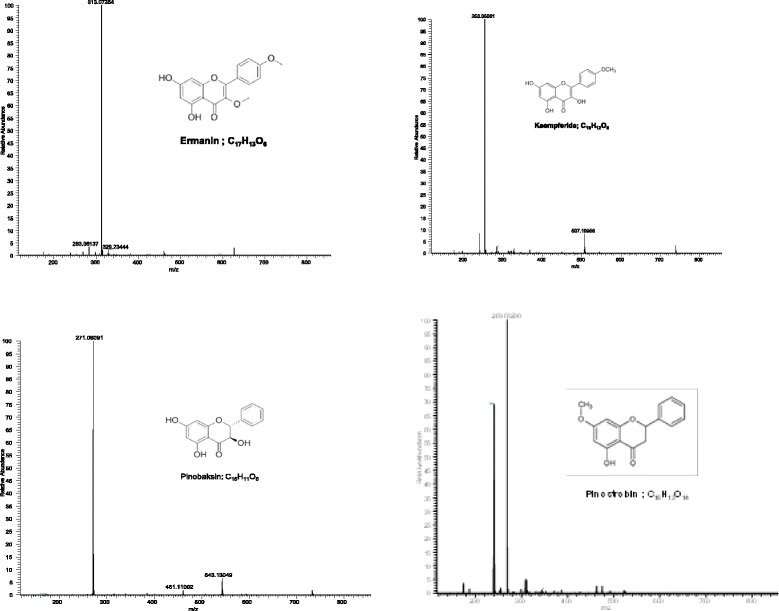
MS spectra and structure of ermanin, kaempferide,pinobaksin andpinostrobin detected in the *Muntingia calabura* ethyl acetate fraction

EAF was analysed based on the accurate mass data of the molecular ions, in which ions detected were tentatively identified by their generated molecular formula, through the software Data analysis (Xcalibur) which provided list of possible elemental formulas, together with the use of standard when available and after thorough survey of the literature*.* The widely accepted accuracy threshold for confirmation of elemental compositions was established at 5 ppm. The UHPLC-ESI analysis of EAF revealed the presence of 22 phenolic compounds (Table 2) which list the peak number, retention time, observed *m/z*, the generated molecular formula and the proposed compound detected. Figure 2 corresponds to the base peak chromatogram in negative ion, with the molecule structure of ermanin, kaempferide, pinobaksin and pinostrobin in Fig. 3.

**Table 2:** Phenolic compounds identified in EAF by UHPLC-MS

Peak No	t^R^ (min)	[M-H]-	Error (ppm)	Formula	Identification
1.	0.45	169.01376	3.433	C_7_H_5_O_5_	Gallic acid
2.	2.34	163.03978	4.964	C_9_H_7_O_3_	Protocatechuic acid
3.	3.10	193.05020	3.443	C_10_H_9_O_4_	Ferulic acid
4.	4.53	599.10547	3.879	C_28_H_23_O_15_	Quercitrin-2″-O-gallate
5.	4.93	939.11377	4.220	C_41_H_31_O_26_	Pentagalloyl-hexoside II
6.	5.05	447.09421	4.523	C_21_H_19_O_11_	Kaempferol-3-*O*-galactoside
7.	5.31	317.0308	5.130	C_15_H_9_O_8_	Myricetin
8.	6.20	193.08661	3.569	C_10_H_9_O_4_	Isoferulic acid
9.	6.91	583.11053	3.941	C_28_H_23_O_14_	Afzelin-O-gallate
10.	7.35	301.03586	4.023	C_15_H_9_O_7_	Quercetin
11.	7.42	603.07928	3.894	C_30_H_19_O_14_	Quercetin dimer
12.	7.67	255.06636	4.605	C_15_H_11_O_4_	Pinocembrin
13.	8.14	593.13116	3.697	C_30_H_25_O_13_	Kaempferol-3-*O*-glucoside
14.	8.18	315.05196	6.478	C_16_H_11_O_7_	Rhamnetin
15.	8.55	271.06094	3.099	C_15_H_12_O_5_	Pinobaksin
16.	8.94	285.04037	3.528	C_15_H_9_O_6_	Kaempferol
17.	10.80	253.05063	4.326	C_15_H_9_O_4_	Chyrsin I
18.	11.67	253.05099	5.749	C_15_H_9_O_4_	Chyrsin II
19.	11.91	299.05597	3.195	C_16_H_11_O_6_	Kaempferide
20.	12.56	313.07245	5.703	C_17_H_13_O_6_	Ermanin I
21	12.78	313.07230	5.224	C_17_H_13_O_6_	Ermanin II
22.	13.32	269.08194	4.105	C_16_H_13_O_4_	Pinostrobin

### Pylorus ligation-induced gastric lesion

**Fig. 4:**
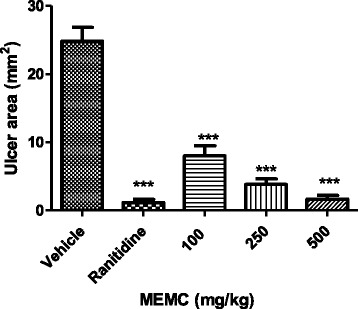
Effect of oral administration of vehicle (Tween 80, 8 %), ranitidine (100 mg/kg) or EAF (100, 250, and 500 mg/kg) on pylorus ligation-induced ulcer. The ulcerated area (mm^2^) was expressed as Mean ± SEM for 6 animals. One way ANOVA was followed by Dunnett's *post hoc* test, ***p < 0.001 *vs.* vehicle

As reported in Fig. 4, EAF at the doses of 100, 250, and 500 mg/kg exerted gastroprotective effect by preventing the development of gastric lesion in a dose related manner. Oral administration of EAF at the abovementioned doses decreased lesion area significantly by 8.0 ± 1.5, 3.8 ± 0.8, and 1.7 ± 0.6 mm^2^, respectively, affording 67.8, 84.6 and 93.3 % protection in comparison to the control, 24.8 ± 2.0 mm^2^ (*p* <0.001). Ranitidine (100 mg/kg), the standard drug used as the positive control in the study significantly inhibit the formation of gastric lesion by 1.2 ± 0.4 mm^2^ (95.3 % protection) as compared to the control group.

### Evaluation of gastric juice parameters

Table 3 shows the effects of EAF upon baseline acid secretion collected after 6 h of pylorus ligature in rats. EAF, at all the three doses (100, 250, and 500 mg/kg) significantly decreased the volume of gastric secretion by 56 % (*p* <0.01), 76 % (*p* <0.001) and 48 % (*p* <0.01), respectively. All the doses of EAF augmented the pH value eventhough they were not significantly different as compared to the negative control. On the other hand, the free and total acidity of EAF reduced significantly at the doses of 250 and 500 mg/kg by about 39 % (*p* <0.001) and 25 % (*p* <0.05), respectively, for free acidity and 40 % (*p* <0.001) and 20 % (*p* <0.05), respectively, for the total acidity. Meanwhile, the lowest dose of EAF (100 mg/kg) failed to significantly decrease the free and total acidity of the gastric secretion. Besides, oral administration of EAF in all the three doses also resulted in significant reduction in total protein content present in the gastric secretion by 21 % (*p* < 0.01), 15 % (*p* < 0.05), and 17 % (*p* < 0.05), respectively, in comparison to the control group. Ranitidine (100 mg/kg), the reference drug used in the assay caused a reduction in the volume of gastric secretion by 71 % (*p* < 0.001), increased the pH by about 2.2 folds (*p* < 0.001), decreased the free and total acidity of the gastric juice by about 49 % (*p* < 0.001) and reduced the total protein content by 31 % (*p* < 0.001) as compared to the control group. The EAF showed a dose-dependent gastroprotective activity as the highest tested dose (500 mg/kg EAF) was significantly more effective in protecting the gastric wall as compared to the lowest dose (100 mg/kg EAF). In contrast, EAF showed no dose response in the antisecretory activity as the 250 mg/kg dose showed maximum reduction in volume of gastric juice. Besides, there was also a significant difference of free and total acidity when 100 mg/kg EAF was compared to 250 mg/kg EAF, but not 500 mg/kg EAF.

**Table 3:** Effect of EAF on gastric juice parameters in pylorus-ligated rat model

Treatment	Volume of gastric juice (ml)	pH	Free acidity (meq/l)	Total acidity (mEq/l)	Total protein (mg/ml)
Control (8 % tween 80)	7.75 ± 1.18	1.26 ± 0.08	103.30 ± 7.53	142.50 ± 9.69	5.45 ± 0.17
Ranitidine	2.25 ± 0.38***	2.73 ± 0.38**	52.67 ± 5.70***	80.67 ± 7.87***	3.78 ± 0.28***
EAF 100 mg/kg	3.38 ± 0.86**	1.97 ± 0.31	97.00 ± 8.42	128.70 ± 4.22	4.32 ± 0.31**
250 mg/kg	1.83 ± 0.23***	1.94 ± 0.20	62.83 ± 7.08***	99.00 ± 6.46***	4.61 ± 0.10*
500 mg/kg	4.02 ± 0.64**	1.69 ± 0.25	77.50 ± 3.43*	115.20 ± 4.80*	4.50 ± 0.13*

Values are expressed as mean ±  SEM for six animals in each group. One way ANOVA was followed by Dunnett's *post hoc * test, *p < 0.05, **p < 0.01, and ***p < 0.001 as compared to the control group

### Determination of mucus in the gastric mucosa

**Fig. 5:**
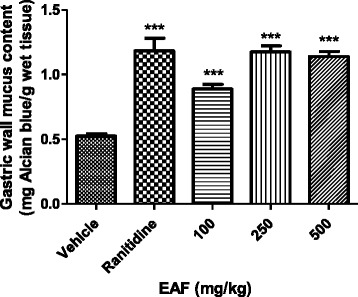
Effect of oral administration of vehicle (Tween 80, 8 %), ranitidine (100 mg/kg) or EAF (100, 250 and 500 mg/kg) on gastric wall mucus produced in the stomach. The gastric wall mucus content (mg Alcian blue/g wet tissue) was expressed as Mean ± SEM for 6 animals. One way ANOVA was followed by Dunnett's *post hoc * test, ***p < 0.001*vs.* vehicle

Pre-treatment of EAF significantly augmented the gastric wall mucus content in all the doses administered (100, 250, and 500 mg/kg) as observed in Fig. 5. Oral administration of the extract and ranitidine increased the mucus content significantly (*p* <0.001) when compared with the control animals pre-treated with vehicle alone.

### Effect of L-NAME and NEM pre-treatment in EAF gastroprotection

**Fig. 6:**
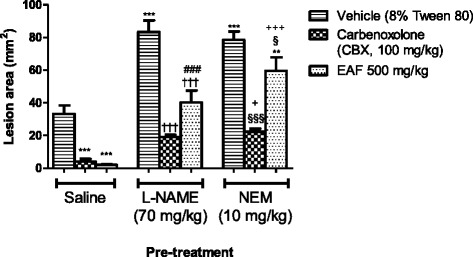
Effect of vehicle (Tween 80, 8 %, p.o.), carbenoxolone (CBX, 100 mg/kg, p.o.) and EAF (500 mg/kg, p.o) on gastric lesions induced by absolute ethanol in rats pre-treated with saline i.p., L-NAME (70 mg/kg, i.p.) or NEM (10 mg/kg, i.p.). Each column represents the Mean ± SEM of 6 animals. One-way ANOVA followed by Newman–Keuls test, **p < 0.01,***p < 0.001 *vs.* saline vehicle; †††*p* <0.001 groups pre-treated with L-NAME *vs.* vehicle L-NAME; §§ *p* < 0.01, §§§ *p* <0.001 groups pre-treated with NEM *vs.* vehicle NEM; ###*p* <0.001 saline pre-treatment vs. its corresponding L-NAME pre-treatment; +*p* < 0.05, +++*p* < 0.001 saline pre-treatment vs. its corresponding NEM pre-treatment

In order to determine if NO and SH compounds are likewise involved in the mechanism of action of EAF, the animals were pre-treated with L-NAME (70 mg/kg) or NEM (10 mg/kg), respectively. Pre-treatment with L-NAME or NEM aggravated the gastric lesions significantly (*p* <0.001) as compared to the vehicle in the saline group. The administration of L-NAME or NEM significantly increased the effects of ethanol on gastric mucosa injury, resulting in the loss of the gastroprotection of EAF (Fig. 6). These results suggested that the gastroprotective effect of EAF implicated a strong participation of the NO and SH compounds that are relevant in the mucosal protection against harmful injuries

### Effect of EAF on SOD, GSH, MDA levels and CAT activity in the stomach tissue of the ethanol-treated rats

The effect of EAF on SOD, GSH, MDA levels and CAT activity in ethanol-treated stomach tissue homogenates is presented in Table 4. Oral administration of ethanol decreased their gastric SOD and GSH levels and CAT activities significantly. The depleted levels or activity of SOD, GSH and CAT were restored significantly by EAF (100, 250 and 500 mg/kg) and ranitidine treatments, parallel to the normal group. On the other hand, there was a significant increase in the gastric MDA content of the ethanol treated group of rats when compared with the normal untreated rats. Treatments of EAF at the doses of 100, 250 and 500 mg/kg to ethanol treated rats decreased the elevated MDA levels significantly; similar to the standard ranitidine treatment, and comparable to the normal rats. Thus, the observation indicated that EAF might attenuate the changes caused by ethanol via regulation of oxidant–antioxidant balance.

**Table 4:** Effect of EAF on levels of SOD, GSH, CAT and MDA in the stomach tissue of the ethanol-treated rats

Experimental groups	Dose (mg/kg)	SOD (U/mg protein)	Total GSH (mM/ mg protein)	CAT (U/ mg protein)	MDA (ng/mg protin)
Normal (untreated)	-	4.90 ± 0.21**	43.35 ± 2.02***	60.59 ± 0.87*	3.11 ± 0.14***
Vehicle + ethanol	-	4.00 ± 0.13^##^	16.76 ± 1.73^###^	55.48 ± 0.76^##^	6.66 ± 0.26^###^
Ranitidine + ethanol	100	5.00 ± 0.15**	55.60 ± 1.65***	63.87 ± 0.62***	3.34 ± 0.18***
EAF + ethanol	100	4.74 ± 0.16*	46.48 ± 3.63***	62.85 ± 2.31**	3.19 ± 0.16***
	250	5.41 ± 0.28***	55.72 ± 3.37***	63.34 ± 1.12***	2.92 ± 0.11***
	500	6.22 ± 0.16***	56.04 ± 3.26***	65.61 ± 1.28***	2.86 ± 0.10***

Values are expressed as mean ±  SEM for six animals in each group. One way ANOVA was followed by Dunnett's *post hoc * test, *p < 0.05, **p < 0.01, and ***p < 0.001 as compared to the vehicle group. Student’s *t*-test was performed for comparing normal and vehicle groups, significant at ^##^ *p* < 0.01, and ^###^ *p* < 0.001

**Table 5:** Effect of EAF on levels of PGE_2_ and NO in the stomach tissue of the ethanol-treated rats

Experimental groups	Dose (mg/kg)	Prostaglandin E_2_(pg/mg protin)	NO (μM/g tissue)
Normal (untreated)	-	188.39 ± 4.66**	43.06 ± 4.83**
Vehicle + ethanol	-	159.90 ± 6.49^##^	22.25 ± 1.65^##^
Ranitidine + ethanol	100	186.88 ± 3.39**	45.73 ± 4.22**
EAF + ethanol	100	178.12 ± 5.07	42.61 ± 2.25**
	250	191.94 ± 5.24***	47.25 ± 4.46***
	500	201.07 ± 4.20***	56.47 ± 4.61***

Values are expressed as mean ±  SEM for six animals in each group. One way ANOVA was followed by Dunnett's *post hoc * test, *p < 0.05, **p < 0.01, and ***p < 0.001 as compared to the vehicle group. Student’s *t*-test was performed for comparing normal and vehicle group, significant at^##^ *p* < 0.01

### Effect of EAF on PGE_2_ levels

The decreased level of PGE_2_ (*p* <0.01) in the vehicle group when compared to that of the normal control group indicated that ethanol treatment reduced the PGE_2_ production. Administration of rats with EAF at doses of 250 and 500 mg/kg significantly (*p* <0.001) increased the level of PGE_2_. Table 5 shows that EAF was able to maintain a high PGE_2_ level in the rats despite being treated with ethanol, similar to the control rats. The low dose of EAF, 100 mg/kg, also led to an increment in PGE_2_ level, though the variation did not reach the statistical significance.

### Effect of EAF on the NO level in the stomach tissue of ethanol treated rats

The effect of the plant extracts on NO level in the gastric homogenate was assessed using Griess reagent and expressed as total nitrate/nitrite. The animals that were treated with ethanol showed significant changes, with respective decrease of NO levels (*p* <0.001) as compared to the respective control animals (Table 5). Administration of ranitidine and EAF at all the three doses showed significant increase (*p* <0.05 for ranitidine and 100 mg/kg EAF; *p* <0.001 for 250 and 500 mg/kg EAF, respectively) in NO level in the stomach homogenate, parallel to the control animals. Therefore, this finding proved the presence of NO in the gastroprotection of EAF.

## Discussion

The present study aimed to investigate the gastroprotective mechanism(s) of the most effective fraction of *Muntingia calabura*, EAF. In our previous study, pharmacological evaluation of the three different fractions of *Muntingia calabura* was carried out and we have found that EAF appeared to be the most effective fraction with high antioxidant and anti-inflammatory activities which contribute to the prophylactic effect of the extract against gastric ulceration in rats [30]. Therefore, EAF was preceded to the mechanistic study to determine the underlying gastroprotective mechanism of the fraction. For many decades, gastric secretion is known to be a key factor in gastrointestinal functions and the regulation of acid secretion is important in the pathogenesis of peptic ulcer. In the current study, pylorus ligation induced gastric ulcer model was used to study the effect of the fraction on gastric and mucus secretions. Agents that decrease secretion of gastric acid and/or increase secretion of mucus are effective in preventing the ulcers induced by this method [43]. The ligation of the pyloric end of the stomach causes the accumulation of gastric hydrochloric acid and pepsin, resulting in digestion of the gastric mucosa and breakdown of the gastric mucosal barrier [44, 45]. In order to gain insight into the effects of EAF on gastric secretion as one of the possible mechanism contributing to its gastroprotective actions, the gastric secretion in pylorus-ligated rats were evaluated. Our findings demonstrated that oral administration of EAF significantly reduced the gastric juice volume, free and total acidity in the 6 h pylorus-ligated rats, while exerting significant protection against lesions formed due to accumulation of highly acidic hydrochloric acid. Thus, the possible mechanistic activity of EAF might be attributed to the antisecretory effect, which acts by significantly decreasing the secretion of gastric aggressive factors.

On the other hand, protein content of the gastric juice increased significantly in the ulcer control group while the treatment of EAF resulted in significant reduction in the protein level of the stomach fluid. The elevation in protein content of gastric juice indicates damage to the gastric mucosa caused by plasma protein leakage into the gastric fluid [37]. In addition, disintegration and degradation of glycoprotein moieties may result in minimal quantity of glycoprotein present in the gastric juice. Thus, the reduction in the glycoprotein moieties may be attributed to the decreased activity of defense mechanisms, leading to gastric mucosa damage [46]. Therefore, administration of EAF was able to decrease the plasma proteins leakage into the gastric juice while increasing the glycoprotein content that acts as a coating and protective barrier on the gastric mucosa.

In gastric ulcers, despite of low acid secretion, weakening of mucosal defenses can also lead to severe injury [10]. Therefore, gastric mucus plays a significant role in protecting the gastric walls from the aggressive factors, providing the first line of mucosal protection against luminal acid. A continuous mucus gel-like protective barrier coats the entire gastric mucosa, maintaining a microenvironment at the mucus-mucosa interface at a pH near to 7, while acting as a barrier against luminal pepsin to protect the underlying mucosa from proteolytic digestion [47]. The mucus consists of mucin-type glycoproteins, which are detected by amounts of Alcian blue binding. Our results showed that EAF treatment augmented the amount of adhered gastric mucus as compared to the vehicle. An increase in mucus secretion may be responsible for the gastric cytoprotection by improving the buffering of acid in gastric juice and acting as an effective barrier to the back diffusion of hydrogen ions as well as reducing stomach wall friction during peristalsis and gastric contractions [48]. Thus, it could be postulated that increase in mucus secretion by EAF play an important role in gastric mucosal protection and it could be one of the potential mechanisms of the gastroprotective effect elicited by EAF.

Overall, the results obtained in the pyloric ligature model demonstrated the significant potential of EAF as an effective gastroprotective agent. Treatment of EAF in this model proves to weaken the gastric aggressive factors while improve the cytoprotective factor, which is via reduction in the volume, free and total acidity of gastric secretion and enhanced mucus secretion to strengthen the gastric mucosal barrier, respectively. EAF also augmented the pH value of the gastric fluid eventhough they were not significantly different as compared to the control group.

In order to evaluate the involvement of endogenous NO and SH compounds in the gastroprotective effect of EAF, L-NAME or NEM were pre-treated to rats that were lesion-induced with ethanol. NO is a ubiquitous molecule generated by nitric oxide synthase (NOS) and is involved in a variety of biological processes. In our current study, administration of L-NAME, a non-selective NOS inhibitor, aggravated lesion formation when induced by ethanol. L-NAME inhibits NO synthesis while causing vasoconstriction of several vascular beds and increases systemic blood pressure, resulting in damage to gastric mucosa and its endothelium [49]. Our results show an increase in gastric lesions when NO production is blocked before EAF treatment. Pre-treatment of the animals with L-NAME reversed the gastroprotective effects exerted by EAF against ethanol-induced damage, suggesting that the gastroprotective effect of the EAF could be mediated by the NO pathway. On the other hand, we have also showed the possible involvement of NO in gastroprotection exerted by EAF by determining the NO level in the stomach tissue of the rats induced with ethanol. Ethanol effectively reduced the level of NO in the gastric mucosa as compared to the control group which could be a result of decreased NOS activity that was associated with an increase in the extent of damage. This finding is in accordance with Hajrezaie et al. [50], Nordin et al. [51] and Rouhollahi et al. [52]. Low levels of NO could also be due to the consumption of NO in the free radical reactions, resulting in overproduction of peroxynitrites (ONOO^−^) during ethanol metabolism [53]. According to Rouhollahi et al. [52], formation of gastric lesions induced by ethanol is remarkably abolished by NO-stimulating drugs, while a reduction in NO synthesis could increase the susceptibility of the gastric mucosa to the destructive effects of ethanol. In our study, EAF exerted a significant protective mechanism by increasing the levels of NO in the EAF treated stomach tissue. The increased levels of NO play an important role in gastric protection through the dilation of gastric blood vessels. This result in an increased supply of nutrients that contributes to the multiplication of cells that constitute the granulation tissue, which is the first tissue formed in the regeneration process [8]. Thus, the gastroprotective effect afforded by EAF could be associated with the involvement and modulation of NO, which results in protection of gastric mucosa against damage induced by noxious agent such as ethanol.

Furthermore, the administration of NEM, a SH- blocker, significantly increased the effects of ethanol on gastric mucosa injury, reversing the gastroprotective effect of EAF. The absence of SH group following the NEM administration reduces the extract’s ability to exert gastroprotective activity. Endogenous SH compounds play a critical role in maintaining gastric mucosal integrity and they are believed to be the key agents in mucosal protection against ethanol-induced gastric injury. Its continuous adherence to mucus layer is a barrier to luminal pepsin and creates a stable, undisturbed layer to support the surface neutralization of acid. This prevents the proteolytic digestion of the underlying mucosa [47]. SH compound also unites the mucus subunits, forming disulfide bridges, which prevents the mucus from becoming water-soluble and easily withdrawn by ulcerogenic agents, such as ethanol [54]. Besides that, the SH-groups have the ability to bind the free radicals generated by noxious agents and act as recycling antioxidants, thus controlling the production and nature of mucus [6, 55]. Therefore, our results suggest that the gastroprotective effect of EAF involves a strong participation of the SH- compounds, indicating the importance of an intact sulfhydryl barrier that is relevant in the mucosal protection against harmful injuries.

In our previous study, we have proved that EAF was able to markedly ameliorate gastric ulceration in ethanol-induced rats [30]. Hence, in our current study, the mechanisms involving enzymatic and non-enzymatic systems including SOD, CAT, GSH and MDA, have been investigated and their involvements are proven. Ethanol administration increases lipid peroxidation, decreases SOD, CAT, GSH levels and the protective factors of the gastric mucosa. SOD, CAT and GSH, are the mutually supportive team of antioxidant enzymes, providing an effective defense system against ROS. SOD is considered to be the first line of defense against the deleterious effect of ROS in cells. It catalyzes the dismutation of superoxide radical (O^−^) to either ordinary molecular oxygen (O_2_) or hydrogen peroxide H_2_O_2_ [56]. In our study, administration of ethanol was shown to inhibit SOD activity. This may cause an interruption in conversion of the O^−^ radicals resulting in an increased flux of O^−^ in cellular compartments [57]. This may cause an increase in oxidative degradation of lipids, known as lipid peroxidation, where the O^-^ radicals may attract electrons from the lipids in cell membranes, resulting in cell damage. Hence, the increased lipid peroxidative indices in our current study that was proved via the MDA assay could be associated with the reduced SOD levels. MDA is the final product of lipid peroxidation. It is widely used as a marker to determine the level of lipid peroxidation [51]. Lipid peroxidation occurs when the activated ROS attacks the unsaturated fatty acids of cell membrane phospholipids, causing damage to the membrane phospholipid, leading to cell injury [58]. In our present study, an increment in MDA level was observed in the stomach of ethanol-ulcerated rats. This indicates the enhanced lipid peroxidation and over production of free radicals, which results in tissue damage and failure of the antioxidant defense mechanisms to inhibit excessive free radicals formation. Oral administration of EAF was able to significantly reverse these changes and markedly reduced concentration of MDA, thus suggesting that the mechanism of gastroprotection of EAF could be due to its antiperoxidative potential by virtue of its free radical scavenging activity. Meanwhile, CAT is a heme-protein, which catalyzes the reduction of H_2_O_2_ and protects the tissue from highly reactive oxygen free radicals and hydroxyl radicals [9]. CAT also acts as preventive antioxidant that triggers the rapid conversion of peroxyl radical into biologically safe substances, such as water [59]. CAT activity was increased in the EAF or ranitidine treated rats, but the activity was decreased when given ethanol alone. A reduction in CAT level may interrupt the degradation of H_2_O_2_ that was produced from the dismutation of O^-^ radicals by SOD. On the other hand, GSH, the non-enzymatic biological antioxidant found abundantly in the gastric mucosa of humans and rats, plays an important role in removing H_2_O_2_, superoxide anions and alkoxy radicals with a consequence of attenuating tissue damage [9]. GSH also helps in maintaining the mucosal integrity. The depletion of GSH is associated with the accumulation of highly reactive free radicals, which results in loss of function and integrity of cell membranes [60]. Hence, the reduction in GSH level in group that received only ethanol and the reversal effect of EAF or ranitidine suggest that the gastroprotective activity of EAF may appear through participation of GSH. On the whole, the oral administration of EAF normalized gastric mucosal levels of SOD, MDA, CAT and GSH in response to oxidative stress caused by ethanol. Thus, our findings clearly demonstrate the significant role played by the antioxidant actions of EAF in counteracting the detrimental effect evoked by ethanol in the digestive system.

Furthermore, the role of PGE_2_ in mediating the gastroprotective effect of EAF was also investigated. The level of PGE_2_ dropped in the ulcer control group, as administration of ethanol may reduce the synthesis of PGE_2_ in gastric mucosa. However, the level of PGE_2_ was elevated following the treatment of EAF, suggesting that the gastroprotective effect of EAF could be mediated by PGE_2_. In the stomach, prostaglandins play a vital protective role by improving blood flow to maintain the cellular integrity in the mucosa, stimulate mucus secretion and enhance secretion of bicarbonate and SH compounds to strengthen the resistance of gastric mucosal cells to the necrotizing effect of strong irritants [1, 61]. In addition, prostaglandins also help to regulate mucosal cell turnover and repair, while suppressing the aggressive factors, such as acid and pepsin secretion [62]. Besides that, according to Heeba et al. [63], the prostaglandins influence virtually every component of the mucosal defense, which includes inhibiting leukocyte recruitment and enhancing the resistance of epithelial cells against potential damage by cytotoxins. The increase in mucosal generation of PGE_2_ observed after treatment with EAF probably mediated, at least in part, by NO. As reported above, the gastroprotective activity of EAF possibly involves the modulation of NO and NO has been reported to increase PGE_2_ biosynthesis through a cyclic guanosine monophosphate independent-mechanism. Hence, it could be assumed that NO might involve in the regulation of PGE_2_ biosynthesis and/or release in the stomach after damage [64].

The gastroprotective potential of EAF could also be attributed to the presence of quercetin and gallic acid, from the class of flavonoid and phenolic acid, respectively. In the present study, we have showed the presence and quantified both quercetin and gallic acid in EAF. Quercetin is the most powerful flavonoid, which affords protection against ROS and reactive nitrogen species (RNS) produced during the normal oxygen metabolism or induced by exogenous damage in the body [65]. Quercetin is widely studied and has been shown to possess significant antiulcer and gastroprotective activity and has been found to protect gastric mucosa against ethanol, acetic acid, acid ethanol, ischemia/reperfusion, stress, pylorus ligation, reserpine, indomethacin and *Helicobacter pylori* [7, 66–72]. The best described property of quercetin is its ability to act as antioxidants, which include enzymatic and non-enzymatic antioxidants that control the level of ROS/RNS and repair oxidative cellular damages. The antioxidant mechanisms involved in the gastroprotective effect of quercetin include inhibition of lipid peroxidation, reduction of protein carbonyl compounds and increase in the SOD activity as well as enhance the levels of mucosal non-protein SH compounds in gluthathione peroxidase activity [67, 68, 73]. Hu et al. [74], quercetin was reported to protect H_2_O_2_-induced oxidative damage in human gastric epithelial cells and ameliorate ROS production in acute gastric injury in mice. This clearly supports our findings of increased SOD and GSH levels and reduced MDA activity in the stomach homogenate treated with EAF. Besides, quercetin is able to inhibit acid production in isolated parietal cells in response to histamine and dibutyryl-cAMP stimulation and also acts in preventing H^+^/K^+^-ATPase activity [75]. Hence, the gastroprotective effect of quercetin could be due to its antiperoxidative, antioxidant and antihistaminic effects.

On the other hand, gallic acid is a naturally abundant polyhydroxy phenolic compound consumed as dietary herbal supplement [76]. Sen et al. [77] have showed that gallic acid exerts its antiulcer effect in aspirin plus pylorus ligation-induced gastric lesion by attenuating the offensive factors and increasing the mucosal defensive factors, while activating the antioxidant mechanisms and inhibiting toxic oxidant mechanisms in stomach tissues. Another recent study by Abdelwahab [78] revealed the gastroprotective mechanism of gallic acid in ethanol-induced ulcerogenesis. Abdelwahab has claimed that the major mechanism of action of gallic acid as an antiulcer agent is due to their effects on gastric acid secretion, promotion of mucosal protection by endogenous factors (NO, PGE_2_ and tumor necrosis factor-α), inhibition of oxidative stress-induced apoptosis, prevention of proinflammatory cytokines production and inhibition of histamine release from mast cells. Yen et al. [79] has reported the excellent antioxidant activity of gallic acid. Therefore, it is plausible to suggest that the gastroprotective activity of PEF, EAF and MEMC could be due to the presence of quercetin and gallic acid. However, EAF showed to contain the highest amount of quercetin and gallic acid, followed by MEMC and PEF, which may explain the reason why EAF stands out to be the most effective fraction affording its gastroprotective activity as compared to the other extracts.

The UHPLC-ESI analysis of *Muntingia calabura* fraction proved the presence of quercetin and gallic acid via the MS analysis. In addition, we have also detected the presence of 20 other compounds in the fraction. Among them, pinocembrin, pinostrobin, pinobaksin, chyrsin, and ermanin has been reported previously to be present in the leaves of *Muntingia calabura* [80]. Meanwhile, we have revealed, for the first time, the presence of few other flavonoids, which could contribute to the gastroprotective action of *Muntingia calabura*. They include protocatechuic acid, ferulic acid, quercitrin-2″-O-gallate, pentagalloyl-hexoside II, kaempferol-3-O-galactoside, myricetin, isoferulic acid, afzelin-O-gallate, kaempferol-3-O-glucoside, rhamnetin, kaempferol and kaempferide. Protocatechuic acid is a type of widely distributed naturally occurring phenolic acid, which is similar to gallic acid, caffeic acid, vanillic acid, and syringic acid that are well-known antioxidant compounds. Protocatechuic acid ethyl ester was found to possess significant antiulcer property in ethanol, aspirin or pyloric-ligation induced gastric lesions in rats. The mechanism of action of protocatechuic acid ethyl ester may be due to either cytoprotective action of the drug or by strengthening the gastric mucosa thereby enhancing mucosal defense [81]. Similar to several other phenols, ferulic acid also exhibits antioxidant activity in response to free radicals, resulting in strong anti-inflammatory activity [82]. A very recent study showed that quercitrin, afzelin and isoferulic acid were able to significantly reduce gastric ulceration in HCl/ethanol-induced rats as these compounds are able to act as free radical scavengers and some of them were found to increase the mucosal content of prostaglandin in tissues, affording protection to the gastric mucosa against various ulcerogens. Besides, isoferulic acid was also reported to possess strong antioxidant properties while able to effectively inhibit lipid peroxidation [83]. Furthermore, quercitrin 2′-O-gallate were reported to exhibit moderate to strong radical scavenging properties on lipid peroxidation, hydroxyl radical, superoxide anion generation and DPPH radical [84]. Kaempferol has been reported to exert significant antiulcer activity in ethanol-, stress- and pylorus-ligation-induced ulcer models while myricetin was found to be active in reserpine- and stress-induced ulcer models [7]. Kaempferol causes a decrease in acid-pepsin secretion and increase in mucin secretion, while increasing endogenous prostaglandins and decreasing leukotrienes [85].

## Conclusion

In conclusion, our current findings suggest that EAF exerts its gastroprotective activity via several mechanisms. The antisecretory effect via reduction in the volume, free and total acidity and the strengthening of gastric mucosal barrier via enhancement in mucus secretion suggests the balanced protection of EAF against the aggressive and defensive factors of gastric ulcer. Another underlying mechanism of gastroprotection of EAF may involve the modulation of NO and SH compounds, which are two important components that play important role in gastric mucosal protection. Besides, the gastroprotective activity of EAF against oxidative injuries caused by ethanol is most likely due to its strong antioxidant and antiperoxidative properties as EAF augments the activities of CAT, SOD and GSH, and attenuates the MDA level in stomach tissues, respectively. Moreover, an increase in PGE_2_ level suggests that the protective effect of EAF could also be mediated by PGE_2_. The presence of various flavanoids found in EAF could explain the effectiveness and the best activity of this fraction in affording protection against gastric damages. Therefore, our experimental findings render EAF as a promising constituent for the protection from gastric ulcer. Furthermore, the elucidation of the underlying mechanisms of action also places the traditional use of *Muntingia calabura* leaves in gastroprotection on a solid scientific footing.
